# Outcomes of Catheter-Delivered Thrombolytic Therapy and Resuscitative Measures in a Cardiac Arrest Patient With Massive Pulmonary Embolism: A Case Report

**DOI:** 10.7759/cureus.28654

**Published:** 2022-08-31

**Authors:** Chafika Lasfer, Manal Yaslam, Zebunnisa Sohail

**Affiliations:** 1 Emergency Medicine, Fakeeh University Hospital, Dubai, ARE; 2 Emergency Medicine, Rashid Hospital, Dubai, ARE

**Keywords:** pe thrombolysis, acute pulmonary embolism, venous thromboembolsim, emergency medicine, resuscitation

## Abstract

Acute massive pulmonary embolism is the most critical presentation of venous thromboembolism that needs early detection and management for a better outcome. We present the case of a 42-year-old female who presented to the emergency department (ED) complaining of acute dyspnea and descended into cardiac arrest. Working through the advanced cardiac life support guidelines and appropriate resuscitative measures, having high clinical suspicion supported by bedside ultrasound findings, massive pulmonary embolism was the most likely diagnosis, and so the patient was treated with thrombolytic therapy delivered via a central venous catheter. Return of spontaneous circulation was achieved, and consequently, she made a complete recovery with no adverse neurological or hemodynamic sequelae.

The aim of presenting this topic is to review the literature available on approaches to thrombolytic doses in life-threatening cases of massive pulmonary embolism and to add to an already ongoing discussion about the effects and outcomes of various dosing regimens.

The above facts will lead us to conclude that any discussion seeks to remind us of the primary management principle. All physicians should bear this in mind while managing any case ("*primum non-nocere*," which is a Latin phrase that means "first, do no harm"); it helps to fuel ideologies to seek best practice interventions that ensure the best outcome for pulmonary embolism patients. And such experiences are worth sharing with the world.

## Introduction

With an overall annual incidence of 100-200 per 100,000 inhabitants, acute massive pulmonary embolism is the most critical presentation of venous thromboembolism with a high mortality rate [1]. However, with all the advances in emergency medicine, diagnosing and treating pulmonary embolism remains challenging. Therefore, we aim to highlight the importance of using adjuncts such as point-of-care ultrasound in cardiac arrest cases and to shed light on the dosing and mode of delivery of thrombolytics and their effect on the overall treatment outcome.

## Case presentation

A 42-year-old female was brought by ambulance to our emergency department (ED). She had complained of shortness of breath for three days, which increased on the day of admission, accompanied by palpitations, sweating, and dizziness. She initially went to another hospital and was prescribed propranolol 40 mg for unexplained palpitations and tachycardia, which she took three hours before the presentation on the same day. Following consumption of the beta-blocker, she developed sweating and dizziness.

On arrival, she was conscious, diaphoretic, and tachypneic. Her vital signs on arrival were as follows: temperature 36^o^C, pulse 44/min, respiratory rate 26/min, SpO_2_ 82%, and blood pressure 83/64 mmHg. Her airway was maintained. Chest auscultation revealed harsh breathing sounds bilateral. Heart sounds on auscultation were normal, the abdomen was soft, and there was no evidence of peripheral edema. Her past medical history was positive for deep vein thrombosis in 2006, with no other significant comorbidities. She had no surgeries in the past and no other identifiable risk factors for pulmonary embolism.

**Figure 1:**
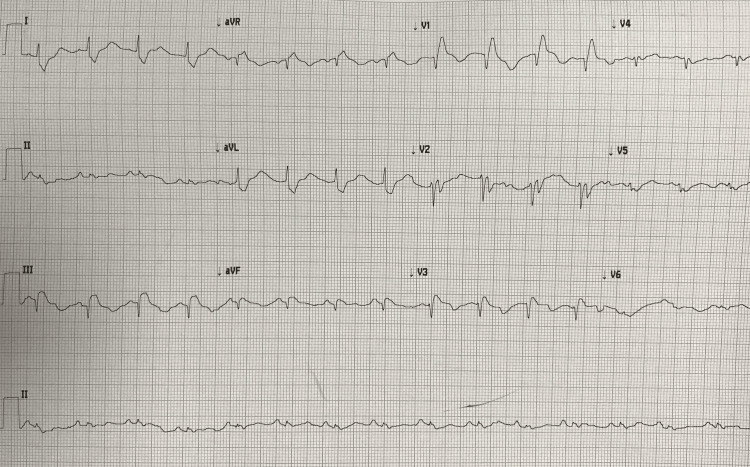
Patient’s ECG showing regular, sinus rhythm, with right bundle branch block prolonged QRS

The patient received 0.5 mg of Atropine during her initial assessment, but she became more bradycardic (pulse 29/min). She had one intravenous line available initially; a second intravenous line was unattainable. The patient descended into pulseless electrical activity, so cardiopulmonary resuscitation (CPR) was initiated per advanced cardiac life support guidelines (ACLS). Intubation was performed using succinylcholine and etomidate, and a right femoral vein central venous catheter was inserted as second intravenous access could not be achieved. Return of spontaneous circulation was achieved within two minutes. A 12-lead electrocardiograph (ECG) was ordered (Figure 1) and showed normal sinus rhythm with right bundle branch block; no evidence of myocardial infarction was noted. The patient was started on post-intubation medication, ventilation settings adjusted, and blood collected for laboratory investigations. The patient had been started on intravenous fluids and a dopamine infusion (10-20 mcg/kg/min) to maintain her circulation. Differentials for pulseless electrical activity were investigated and effectively ruled out during the resuscitation efforts, except for a suggestion of pulmonary thrombosis.

**Figure 2:**
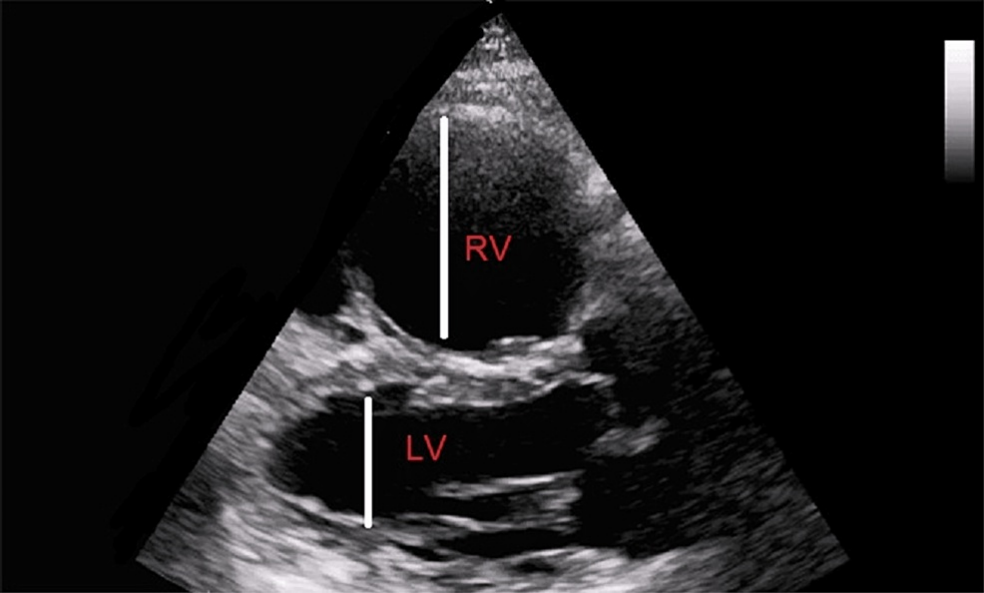
Bedside ultrasound showing dilated RV in comparison to LV RV: Right ventricle; LV: Left ventricle.

Bedside ultrasound showed a dilated right ventricle (Figure 2). Heparin 6400 IU IV was administered. The patient descended into two further episodes of cardiac arrest again; ACLS protocol was initiated each time, and spontaneous circulation was returned three minutes later in each episode. Throughout her time in the ED, the patient experienced numerous episodes of cardiac arrest and pulseless electrical activity each time and received 40 minutes of advanced cardiac life support resuscitative measures. Given the patient's critical presentation of suspected massive pulmonary embolism, the use of intravenous thrombolytics was indicated. Her husband gave consent, and arrangements were made to administer intravenous alteplase. A 100 mg alteplase was administered as boluses of 50 mg each during cardiopulmonary resuscitation via a central venous catheter. The patient developed a small amount of coffee ground aspirate via the nasogastric tube and a small hematoma over the right thigh. Hence, she was started on proton-pump inhibitors (bolus and infusion). The mobile chest x-ray was normal. The patient did not experience any further episodes of cardiac arrest thereafter.

**Figure 3:**
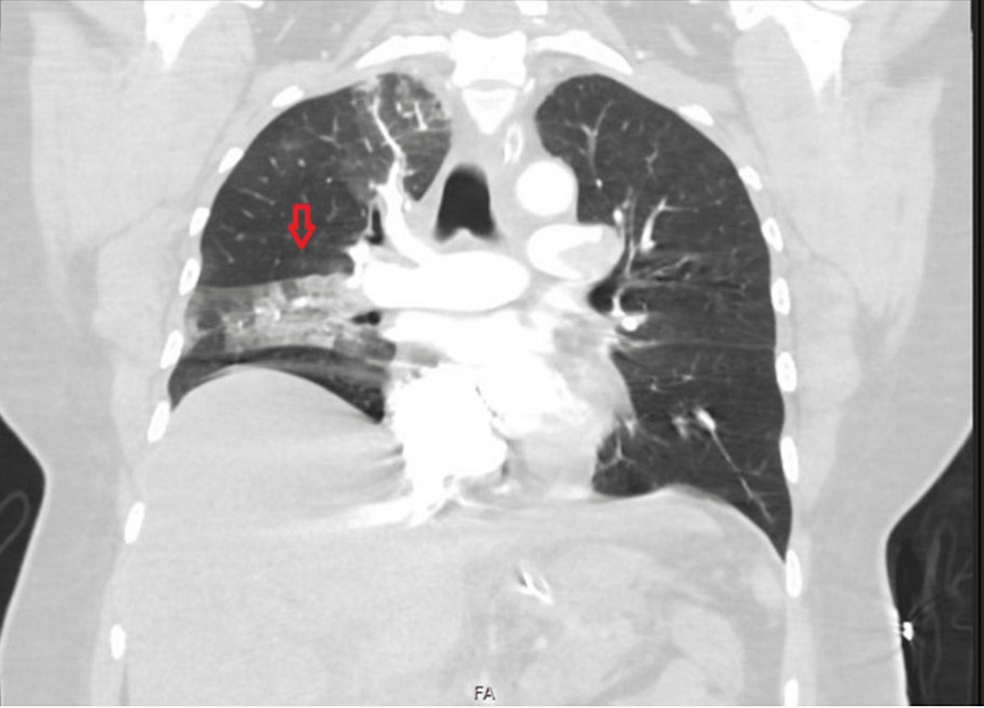
CT-pulmonary angiography: coronal view showing pulmonary emboli with segmental pulmonary infarcts in the right lower lobe

**Figure 4:**
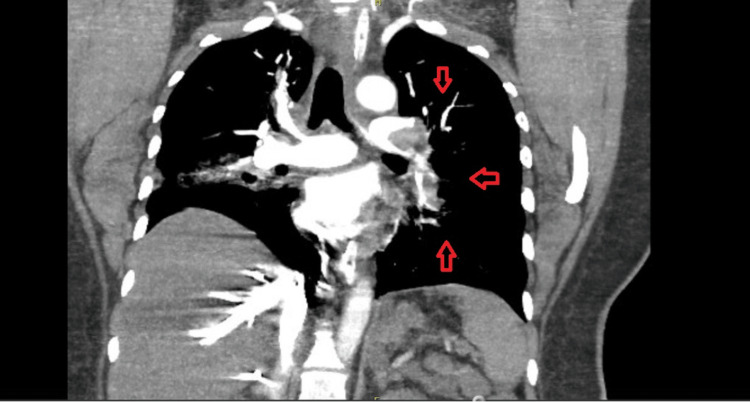
CT-pulmonary angiography: arterial phase revealing a thrombus causing significant luminal stenosis of the left main pulmonary artery

**Figure 5:**
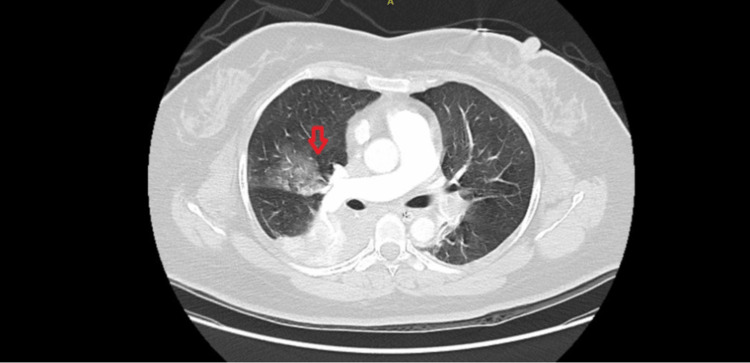
CT-pulmonary angiography: sagittal view showing consolidation in the lateral segment of the right middle lobe with volume loss suggestive of infarcts

Having been thrombolysed, on a ventilator, with ongoing inotropic support and symptomatic measures, the patient was stabilized. She underwent further diagnostic imaging, including CT brain and CT-pulmonary angiography. CT brain was normal and showed no evidence of cerebral hemorrhage. CT-pulmonary angiography showed evidence of pulmonary emboli with segmental pulmonary infarcts in the right lower lobe (Figure 3), with a hypodense filling defect suggestive of thrombus causing significant luminal stenosis extending from the left main pulmonary artery bifurcation into the upper and lower lobe pulmonary arteries and their proximal segmental branches (Figure 4) along with partial thrombotic occlusion of the segmental branches of the right-sided upper and lower lobar pulmonary arteries. Consolidation was noted in the lateral segment of the right middle lobe with volume loss suggestive of infarcts (Figure 5).

The patient was admitted under the care of cardiology as a case of massive pulmonary embolism. She underwent ultrasound Doppler for her bilateral lower limbs that were conclusive for a non-occlusive peripheral thrombus in both mid and distal femoral veins, with echogenic thrombus and peripheral recanalization in the right proximal common femoral vein and right proximal great saphenous vein.

Her hospital stay lasted for two weeks. She was gradually weaned off the ventilator, sedative, and inotropic measures. She was started on subcutaneous clexane (low molecular heparin) 60 mg and oral anticoagulant - warfarin. Transthoracic echocardiography showed fair left ventricle systolic function, ejection fraction of 50%, trivial mitral and tricuspid regurgitation, no clot/vegetation, intact septae and hypokinesia of distal anterior, and basal and mid septum. She was followed by the gastroenterologist and the medical team for upper GI bleeding (endoscopy showed evidence of moderate erosive duodenitis) and control of comorbidities, such as diabetes and hypertension.

During her stay, the patient's general condition improved until discharge. She was conscious and oriented, had a normal systemic examination, and had an overall stable general condition. She was discharged with advice to adhere to the medications prescribed strictly and to return to ED in case of any chest discomfort, dyspnea, or syncopal episode.

## Discussion

Management strategies surrounding massive pulmonary embolism are still controversial. Although many guidelines agree on the supportive role of intravenous thrombolytics, the recommendations are usually of the Level B category, with none of Level A supporting evidence.

Massive pulmonary embolism is defined as patients with a systolic blood pressure of <90 mmHg for >15 minutes, a systolic blood pressure of <100 mmHg with a history of hypertension, or a >40% reduction in baseline systolic blood pressure [1]. Theoretically, a patient presenting with circulatory collapse and suspicion of pulmonary embolism on ultrasound in cardiac arrest is a textbook indication of intravenous thrombolytic.

Per the 2019 European society of cardiology (ESC), echocardiographic evidence of right ventricular (RV) dysfunction in a highly unstable patient is sufficient to prompt immediate reperfusion without further testing. This decision may be strengthened by the (rare) visualization of right heart thrombi [2]. The most significant benefit is observed when treatment is initiated within 48 hours of symptom onset, but thrombolysis can still be helpful in patients who have had symptoms for six to 14 days [3].

The 2019 ESC guidelines for acute pulmonary embolism management advocate the use of thrombolytic therapy (Level 1) in combination with anticoagulation: unfractionated heparin for a favorable outcome with rapid restoration of pulmonary perfusion (Level 1C) [2]. The American College of Emergency Physicians 2018 guidelines for suspected pulmonary embolism remained unchanged from the 2011 guidelines in the above critical point and gave Level B recommendations [4,5]. In centers fully equipped with a mechanical or surgical thrombectomy setup, such an intervention could be used as an alternative to intravenous thrombolytics. However, in unstable patients with suspected pulmonary embolism, they give only Level C recommendations for consideration of thrombolytic therapy for whom the diagnosis of pulmonary embolism cannot be confirmed promptly [6].

There is also no agreement about the thrombolysis regimen in cases of massive pulmonary embolism, understandably due to the perceived risk of bleeding. Numerous regimens have been studied and tested. Currently, three thrombolytics are approved and tested for use in pulmonary embolism (Alteplase, Streptokinase, and Urokinase) (Table 1). But dosing regimens vary, e.g., alteplase 0.6-1 mg/kg or 100 mg as two 50 mg boluses 30 minutes apart or 15 mg bolus followed by 85 mg over 90 minutes or 100 mg over 15 minutes, and tenecteplase as 50 mg bolus or 0.5 mg/kg bolus.

**Table 1:** Regimens approved for use in pulmonary embolism PE: Pulmonary embolism; FDA: Food and Drug Administration. Source: Reference [8].

	Streptokinase	Urokinase	Alteplase	Reteplase	Tenecteplase
Generation	First	First	Second	Third	Third
Clot-specific?	No	No	Yes	Yes	Yes
Half-life (minutes)	12	7-20	4-10	11-19	15-24
FDA-approved for PE?	Yes	Yes	Yes	No	No

As per the 2019 ESC guidelines for pulmonary embolism, two-hour accelerated regimens are preferable. On the other hand, a meta-analysis published in 2005 found 26 studies regarding the use of alteplase infusion, bolus-dose alteplase, and streptokinase. Capstick and Henry concluded that the search revealed no significant difference between the three regimens, but a scarcity of data compromised it [6]. A broad overview of the study showed that alteplase infusion was more effective than bolus-dose alteplase (relative risk [RR]: 1.95; 95% confidence interval [CI]: 1.19-3.2) and associated with lower mortality risk, whereas streptokinase was more effective than alteplase infusion (RR: 1.27; 95% CI: 1.09-1.47) [2,7]. But overall, most studies and print material have advocated for the beneficial effects of IV thrombolytics in such critical situations. Most patients ended with a favorable outcome and endpoint, assessed by clinical and echocardiographic improvement within 36 hours.

Newer evidence also has shown that in-hospital mortality attributable to pulmonary embolism was lower in unstable patients who received thrombolytic therapy compared with those who did not (RR: 0.20; 95% CI: 0.19-0.22; p < 0.0001) [8]. There is sufficient evidence in the literature supporting the life-saving role of thrombolytics in cases of treating massive pulmonary embolism. But does dosing play a significant role? Controversy surrounds thrombolytic dosing as well. There is no agreement regarding dosing regimens.

Numerous studies in the literature have been published testing different dosing regimens. A few to name are the studies by Wang [9] and Goldhaber et al. [10]; each studied different dosing regimens of thrombolytic therapy in case of submassive or massive pulmonary embolism. All concluded that there was no significant difference in the endpoint result of the various dosing schemes. The recently published "OPTALYSE PE" (Optimum Duration of Acoustic Pulse Thrombolysis Procedure in Acute Pulmonary Embolism) trial tested four dosing regimens (two different doses over different durations) to assess which regimen had better efficacy in reaching a measurable endpoint/outcome. The results were equivocal in all four cases [11].

There may not be any consensus regarding dosing intravenous thrombolytics, but the literature referenced above points to the benefits and life-saving role of delivering thrombolysis in cardiac arrest patients with pulmonary embolism. Therefore, it is pertinent that emergency physicians make use of the life-saving potential of these drugs. Moreover, given the evidence, albeit based on case reports and series, of positive outcomes of patients who were thrombolysed while in cardiac arrest for massive pulmonary embolism, further research must delve into the matter, focusing on optimal delivery and dosing of the medication.

## Conclusions

Acute massive pulmonary embolism is the most critical presentation of venous thromboembolism with a high mortality rate. Therefore, it is necessary to maintain a high index of suspicion as early diagnosis of pulmonary embolism improves outcomes for all patients, including patients in cardiac arrest. Correctly using available adjuncts in the ED, such as point-of-care ultrasound in cardiac arrest cases, is incredibly beneficial in detecting pulmonary embolism and starting the necessary treatment early. Still, we should remember these are just tools to help support our primary differential diagnosis, and we should rely on the history and physical examination and the holistic approach of the patient.

This report aims to highlight the life-saving features of intravenous thrombolytics. Another noted observation is that the advent of the central venous catheter to deliver the thrombolytics may have contributed to the effectiveness of the treatment. Few studies present to compare the efficacy of peripherally introduced intravenous thrombolytics versus thrombolytics administered via a central venous catheter. It may be the most favorable option for patients with contraindications to thrombolysis and could be considered a less-invasive alternative to surgical embolectomy. It is a venture that would benefit from further research.
